# Lipopolysaccharide challenge significantly influences lipid metabolism and proteome of white adipose tissue in growing pigs

**DOI:** 10.1186/s12944-015-0067-5

**Published:** 2015-07-08

**Authors:** Jun Guo, Zhiqing Liu, Hailin Sun, Yanping Huang, Elke Albrecht, Ruqian Zhao, Xiaojing Yang

**Affiliations:** Key Laboratory of Animal Physiology & Biochemistry, Nanjing Agricultural University, Nanjing, 210095 PR China; Institute of Muscle Biology and Growth, Leibniz-Institute for Farm Animal Biology (FBN), Dummerstorf, Germany

**Keywords:** Acute inflammation, Lipid metabolism, Proteome, White adipose tissue, Pig

## Abstract

**Background:**

White adipose tissue is recognized as a highly active organ, which is closely related to a large number of physiological and metabolic processes besides storing triglycerides. However, little is known regarding the response of adipose tissue to acute inflammation. Therefore, in this study we employed growing pigs to investigate the changes of lipid metabolism and proteome in white adipose tissue after lipopolysaccharide (LPS) stimulation as a model for bacterial infection.

**Methods:**

The expression of lipid metabolism and inflammation related genes was determined by quantitative real-time polymerase chain reaction. Label-free proteomics analysis was used to investigate changes of the protein profile in white adipose tissue and western blot was used to verify changes of selected adipokines.

**Results:**

The results indicated that LPS significantly increased the expression of toll-like receptor (TLR) 2/4 pathway-related genes and pro-inflammatory factors. Lipid metabolism related genes, including acetyl-CoA carboxylase 1 (ACACA), fatty acid synthase (FASN), stearoyl-CoA desaturase (SCD), uncoupling protein 2 (UCP2), and 11 β-hydroxysteroid dehydrogenase type 1 (11β-HSD1), were down-regulated and the lipolytic enzyme activity was decreased after LPS injection. Proteome analysis revealed 47 distinct proteins with > 2-fold changes. The down-regulation of two proteins (cAMP-dependent protein kinase type II-alpha regulatory subunit and β-tubulin) has been verified by western blot analysis. In addition, the abundance of two adipokines (adiponectin and zinc-α2-glycoprotein) was significantly increased after LPS injection.

**Conclusion:**

In conclusion, LPS challenge can cause acute inflammation in white adipose tissue. Concurrently, lipid metabolism was significantly suppressed and the abundance of several proteins changed in white adipose tissue. The results provide new clues to understand the adipose dysfunction during inflammation.

**Electronic supplementary material:**

The online version of this article (doi:10.1186/s12944-015-0067-5) contains supplementary material, which is available to authorized users.

## Background

In the traditional view, white adipose tissue (WAT) in mammals was seen as the main site for energy storage. However, this view changed since more and more studies demonstrated that WAT is not only a passive storage of triglycerides but also a highly dynamic organ which is closely related to a large number of physiological and metabolic processes [1]. WAT actively participates in body energy regulation through a network of endocrine, paracrine and autocrine signals [2]. Recent researches suggest that adipocytes are also immunologically active, and may play an important role in host defense [3–5].

At present, humans and animals frequently suffer from inflammation, which can be induced by bacterial infection, especially by gram-negative bacteria [6]. Obesity, as one of the most serious health risks, is always associated with low-degree inflammation [7]. However, most of the investigations concerning inflammation focus on immunocytes or immune organs [8], only a minority of researchers pay attention to WAT in this regard [9–11]. In those studies, changes in the gene expression profile of human adipose tissue after acute inflammation were investigated [9, 11]. It has been shown that even subacute inflammation induces signs of inflammation and changes in lipoprotein metabolism in the adipose tissue of cats [10]. Meanwhile, it has been demonstrated that fat depots are differently sensitive to lipopolysaccharide (LPS) stimulation as indicated by nuclear factor-κB (NF-κB) activation [12]. LPS is the major component of the outer membrane in gram-negative bacteria [13] and it can cause acute inflammation resulting eventually in all kinds of pathophysiological damages [14]. LPS injection was used in the present study to simulate bacterial infection and to study inflammation-induced changes in lipid metabolism and in the proteome of WAT.

In recent years, following the rapid development of mass spectrometry, proteomics analysis has been frequently used as a very powerful bioanalytical method for solving various scientific problems from medicine, biology and biochemistry [15]. Especially, label-free proteomics analysis has emerged as a high-throughput method for quantitative clinical proteome studies. Therefore, label-free proteomics analysis was used in the present study to determine changes of the protein profile in WAT of growing pigs after LPS stimulation. Together with observed changes in lipid metabolism, the results may provide a promising clue to metabolic and inflammatory responses leading to the development of adipose tissue dysfunction.

## Results

### Expression of genes involved in toll-like receptor 2 and 4 pathway and inflammatory cytokines

**Fig. 1:**
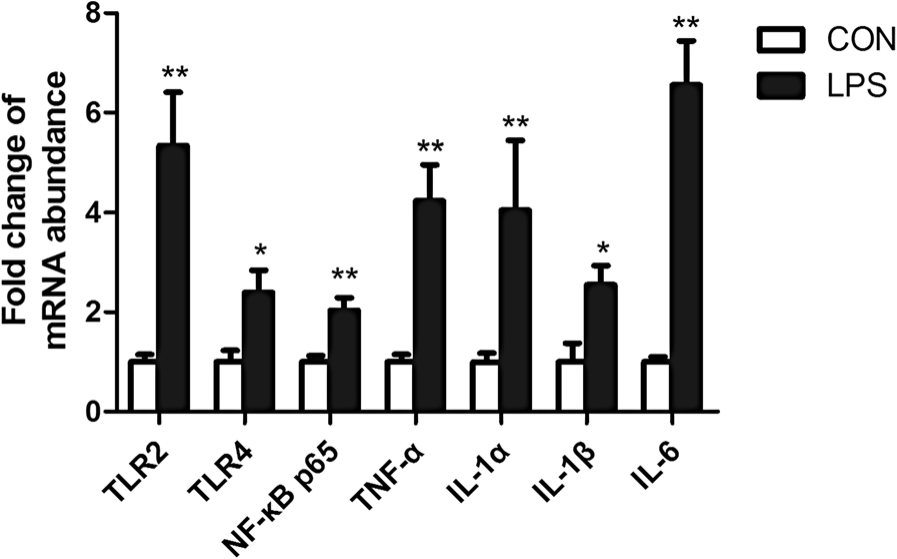
Toll-like receptor pathway and pro-inflammatory factors gene expression after LPS injection compared to control. Data represent the means ± SEM. Data were considered statistically significant when *P* < 0.05, *n* = 6. *represent P < 0.05, **represent P < 0.01

As shown in Fig. 1, the gene expression of toll like receptor 2 (TLR2), TLR4, and NF-κB p65 were all significantly increased after LPS stimulation for 6 h. Similarly, the gene expression of inflammatory cytokines, such as tumor necrosis factor-α (TNF-α), interleukin-1α (IL-1α), IL-1β, and IL-6 was also higher (*P* < 0.05) in LPS stimulated animals compared to controls.

### Expression of genes involved in lipid metabolism

**Fig. 2:**
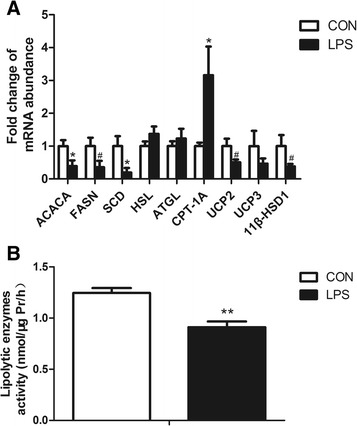
Lipid metabolism gene expression and lipolytic enzymes activity after LPS injection compared to control. **a** Relative mRNA abundance of ACACA, FASN, SCD, HSL, ATGL, CPT-1A, UCP2, UCP3, and 11β-HSD1. **b** Lipolytic enzymes activity. Data represent the means ± SEM. Data were considered statistically significant when *P* < 0.05, *n* = 6. # represent P < 0.1, *represent P < 0.05, **represent P < 0.01

As shown in Fig. 2, the gene expression of key enzymes of lipogenesis, including acetyl-CoA carboxylase 1 (ACACA), fatty acid synthase (FASN), and stearoyl-CoA desaturase (SCD), decreased after LPS treatment (*P* < 0.05). The gene expression of key enzymes of lipolysis, including hormone-sensitive lipase (HSL) and adipose triglyceride lipase (ATGL), remained unchanged (*P* > 0.05). However, as shown in Fig. 2b, the activity of these key enzymes in lipolysis was lower in the LPS injected animals. The gene expression of carnitine palmitoyltransferase-1A (CPT-1A), which is the key enzyme in β-oxidation, was significantly increased in the LPS stimulated group, while the gene expression of uncoupling protein 2 (UCP2) was decreased. The gene expression of 11 β-hydroxysteroid dehydrogenase type 1 (11β-HSD1) showed a trend to lower values in the LPS group.

### Label-free quantitative proteomics analysis

**Fig. 3:**
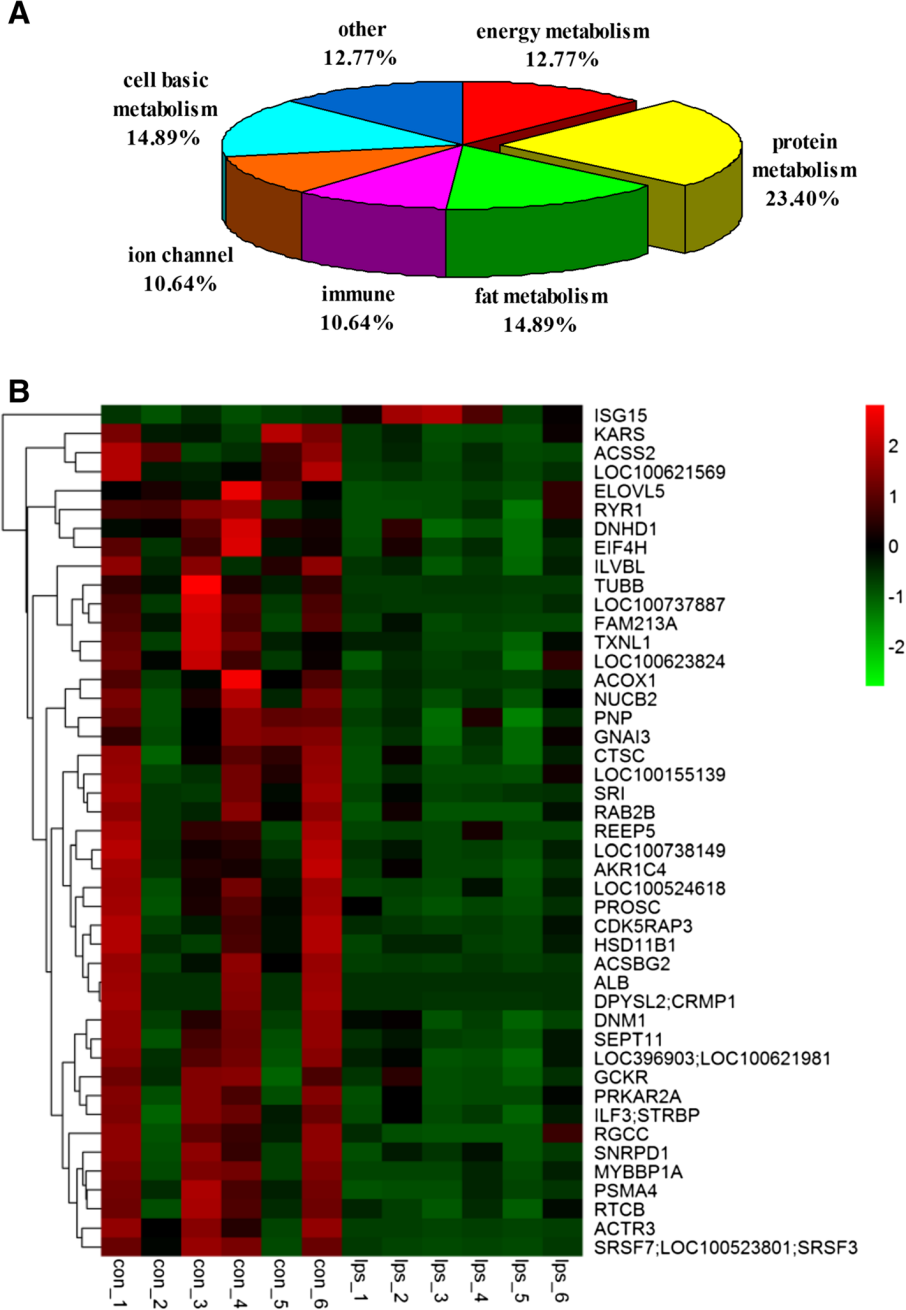
The classification and heat map of distinct proteins in label-free proteomics analysis. **a** The classification of distinct proteins. **b** The heat map of distinct proteins

**Fig. 4:**
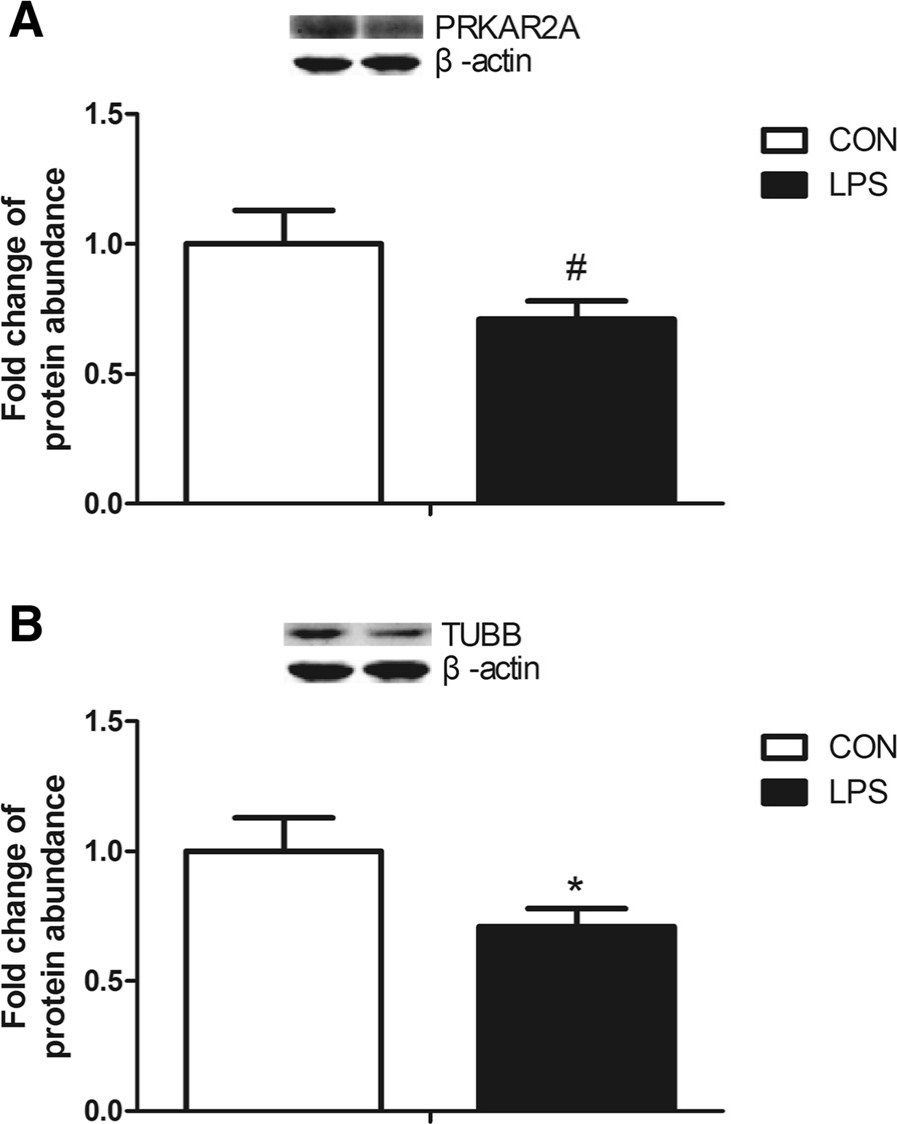
The protein abundance of PRKAR2A and TUBB after LPS injection compared to control. **a** The protein abundance of PRKAR2A. **b** The protein abundance of TUBB. Representative parts of western blots are shown above the graph. Data represent the means ± SEM. Data were considered statistically significant when *P* < 0.05, *n* = 6. # represent P < 0.1, * represent P < 0.05, ** represent P < 0.01

Table 1 specifies 47 proteins which showed at least a 2-fold change between the two groups. The proteins were classified into 7 categories based on their function (Fig. 3a). In Fig. 3b, the heat map of the 47 distinct proteins is shown. The volcano plot and radar chart are shown in Additional file 1: Figure S1 and Additional file 2: Figure S2. The results indicate that cAMP-dependent protein kinase type II-alpha regulatory subunit (PRKAR2A) and β-tubulin (TUBB) were significantly down-regulated following the administration of LPS. The results could be confirmed by western blot analysis (Fig. 4).

**Table 1:** Regulated proteins after LPS injection identified with label-free proteomics analysis

Number	Name	Gene symbol	Protein Name	Difference
1	C1PIG4;P05207	PRKAR2A	cAMP-dependent protein kinase type II-alpha regulatory subunit	down
2	F1RGP1	MYBBP1A	Uncharacterized protein	down
3	F1RI43;P16960	RYR1	Ryanodine receptor 1	down
4	F1RJ89	RGCC	Uncharacterized protein	down
5	F1RLG5	REEP5	Uncharacterized protein	down
6	F1RMP2	DNHD1	Uncharacterized protein	down
7	F1RSP5	LOC100524618	Uncharacterized protein	down
8	F1RUN2;P08835;CON__P02768-1	ALB	Serum albumin	down
9	F1RVZ1	ACOX1	Uncharacterized protein	down
10	F1RWH6	CDK5RAP3	Uncharacterized protein	down
11	F1RX84	PROSC	Uncharacterized protein	down
12	F1RYS5	SEPT11	Uncharacterized protein	down
13	F1S0A2	LOC100737887	Peptidyl-prolyl cis-trans isomerase	down
14	F1S1X9	TXNL1	Uncharacterized protein	down
15	F1S340	SRI	Uncharacterized protein	down
16	F1S458	KARS	Lysine--tRNA ligase	down
17	F1S4Y0	ACSS2	Uncharacterized protein	down
18	F1S8H8	PNP	Uncharacterized protein	down
19	F1S8J6	RAB2B	Uncharacterized protein	down
20	F1S9A4	NUCB2	Uncharacterized protein	down
21	F1S9C9	LOC100155139	Proteasome subunit beta type	down
22	F1SAN6	ILVBL	Uncharacterized protein	down
23	F1SBT8	ACSBG2	Uncharacterized protein	down
24	F1SEQ7	FAM213A	Uncharacterized protein	down
25	F1SF47	HSD11B1	Uncharacterized protein	down
26	F1SNL7;I3LV33	LOC100621569	Uncharacterized protein	down
27	F1STR1	CTSC	Uncharacterized protein	down
28	F2Z528	PSMA4	Proteasome subunit alpha type	down
29	F2Z5D2	ACTR3	Uncharacterized protein	down
30	F2Z5G9	SNRPD1	Uncharacterized protein	down
31	I3L594;I3LRD5	EIF4H	Uncharacterized protein	down
32	I3L5B3;F1SSA6	LOC100621981/MYH10	Uncharacterized protein	down
33	I3L5C8	LOC100623824	Uncharacterized protein	down
34	I3L883	GCKR	Uncharacterized protein	down
35	I3L8P7	LOC100738149	Uncharacterized protein	down
36	I3LAE9;F1RY92;F1S4R5	LOC100523801/SRSF3/SRSF7	Uncharacterized protein	down
37	I3LCZ7;F1RRW8	N/A/DNM1	Uncharacterized protein	down
38	I3LJE2;I3LN38	DPYSL2/CRMP1	Uncharacterized protein	down
39	I3LM05	N/A	Uncharacterized protein	down
40	I3LR71	ELOVL5	Elongation of very long chain fatty acids protein	down
41	I3LU39	ISG15	Uncharacterized protein	up
42	K7GQX0;F1SKU5;K7GM70;K7GL83;F1S585;K7GP99	ILF3/STRBP	Uncharacterized protein	down
43	Q06AS8	GNAI3	GBAK	down
44	Q19PY3;F2Z5V0	RTCB	tRNA-splicing ligase RtcB homolog	down
45	Q2TJA5	AKR1C4	Aldo-keto reductase	down
46	Q767L7	TUBB	Tubulin beta chain	down
47	Q7YQ94;P15981	SLA-DQA1/N/A	MHC class II antigen/SLA class II histocompatibility antigen, DQ haplotype D alpha chain	down

### mRNA and protein levels of selected adipokines

**Fig. 5:**
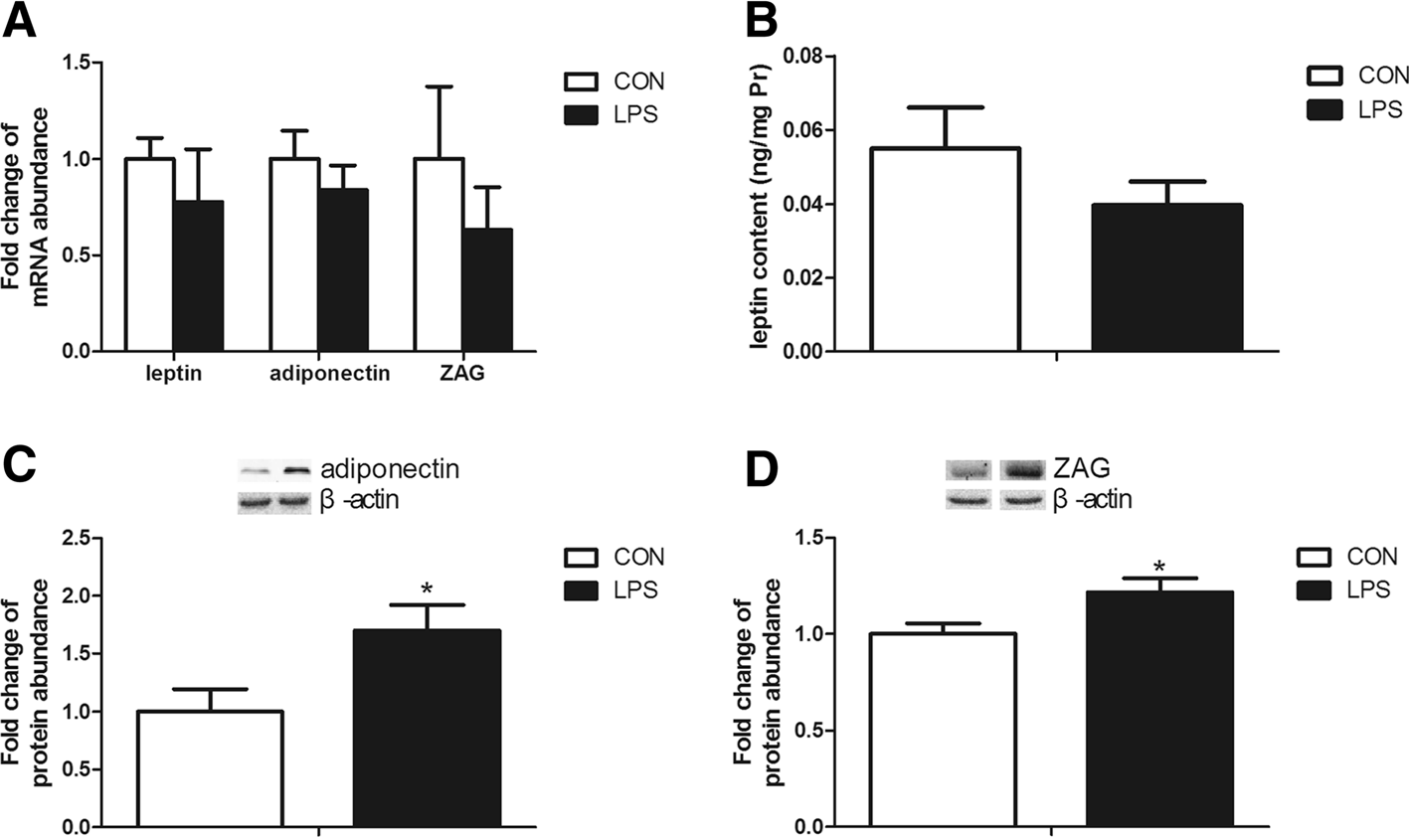
Adipokines gene expression and protein abundance after LPS injection compared to control. **a** Relative mRNA abundance of leptin, adiponectin and ZAG. **b**-**d** Leptin, adiponectin, and ZAG protein abundance. Representative parts of western blots are shown above the graph. Data represent the means ± SEM. Data were considered statistically significant when *P* < 0.05, *n* = 6. # represent P < 0.1, *represent P < 0.05, **represent P < 0.01

As shown in Fig. 5, mRNA abundances of leptin, adiponectin, and zinc-α2-glycoprotein (ZAG) were not different between control and LPS group. Furthermore, the protein content of leptin remained unchanged after LPS treatment. However, the protein levels of adiponectin and ZAG were significantly increased in the LPS group.

## Discussion

Research on inflammation in human and animals usually focuses on immune system, lung, and kidney etc. which are always regarded as targets for drugs to alleviate the inflammation and recover the health [16–22]. Although WAT has already been recognized as a highly active endocrine organ, its response to inflammation is still unclear. Therefore, this study was conducted to investigate changes of lipid metabolism and proteome of adipose tissue after LPS stimulated inflammation in growing pigs. Recent studies have shown that LPS can induce a systemic inflammation [23]. In recognition of the bacterial LPS by host animals and humans, pattern recognition receptors such as toll-like receptors play a critical role in the innate immune system [24]. Ajuwon et al. demonstrated that LPS could induce NF-κB p65 nuclear translocation and increased the expression of IL-6 and IL-15 in pig adipocytes [25]. Our results accordingly showed that LPS activated the TLR pathway, upregulated the expression of pro-inflammatory factors, and eventually resulted in the inflammation of adipose tissue.

It is well known that the metabolic status of WAT itself plays an important role in the process of storing energy. Lipogenesis and lipolysis are two most basic and important processes in lipometabolism of WAT. Key enzymes in the processes of lipogenesis are, among others, ACACA, FASN, and SCD. Furthermore, HSL and ATGL both play an important role in lipolysis [26]. In our study, the gene expression of ACACA, FASN, and SCD was decreased and the activity of the key enzymes of lipolysis (mainly HSL and ATGL) was also depressed after LPS challenge. The results suggest that the acute inflammation, which LPS caused, could suppress metabolic activity in adipose tissue. CPT-1 initiates the translocation of long chain fatty acids across the mitochondrial membranes for beta-oxidation [27, 28]. The uncoupling protein family is a mitochondrial anion carrier family and plays an important role in the biological traits of animal body weight, basal metabolic rate, and energy conversion [29]. After LPS stimulation, although the expression of CPT-1A increased significantly, UCP2 and UCP3 expression was decreased. This result demonstrated that energy expenditure is low in adipose tissue in the condition of acute inflammation. The results proved the effect of LPS on the suppression of metabolic activity.

Our results demonstrated that the expression of a variety of lipid metabolism genes significantly changed after LPS stimulation. Therefore, we employed label-free proteomics analysis to investigate protein changes in WAT. All the distinct proteins were shown in the volcano plot and they were obviously separated from the other proteins. In the radar chart, we can clearly see the extent of change for each distinct protein. According to the results of proteome, from all detected differentially expressed proteins, 23.4 % were related to protein metabolism, 14.9 % to fat metabolism, and 12.8 % to energy metabolism. In general, 51.1 % of the detected proteins were metabolism-related. Based on the results of this study, it is obvious that metabolism in white adipose tissue was seriously affected by LPS stimulation, particularly protein metabolism. These results may help to better understand the relationship between the inflammation and the development of metabolic disorders in white adipose tissue. The proteome data showed that the expressions of acyl-CoA synthetase short-chain family member 2 (ACSS2), acyl-CoA synthetase bubblegum family member 2 (ACSBG2) and ELOVL fatty acid elongase 5 (ELOVL5) were all decreased after LPS stimulation. These three proteins are associated with lipogenesis [30–32]. It is well-known that LPS influences immune response [33]. Accordingly, our results revealed 10.6 % of the regulated proteins were immune-related. It is very interesting to note that the proteins involved in ion channel were also influenced and they account for 10.6 % of regulated proteins. Among the 47 proteins that were identified, the levels of only 1 protein increased, whereas the levels of the remaining 46 proteins decreased significantly following LPS administration. Previous studies have demonstrated that total protein synthesis is reduced in response to adverse conditions, which relieves the burden imposed on the protein quality control system [34]. Additionally, the pausing of ribosomal elongation may be involved [35]. Their results may partly explain why adipose tissue protein decreased in the present study.

Among these detected proteins, 11 β-HSD1 is a key enzyme which can convert inactive glucocorticoid into bioactive forms [36]. Moreover, it is regarded as an important amplifier of glucocorticoid activity in peripheral tissues [37]. Several studies have demonstrated that 11 β-HSD1 is a powerful regulator in modulating WAT metabolism and function [38]. Our results indicated a trend to decreased mRNA expression of 11β-HSD1 in the LPS group and also significantly decreased protein expression. In all eukaryotic cells, microtubules, which are integral components of the cytoskeleton, are highly dynamic long filamentous structures which play a key role in some cellular processes, such as cell division, cell migration and support of cell shape and polarity [39]. Microtubules are protein polymers in the form of hollow cylindrical filaments, composed of α- and β- tubulin heterodimers [40, 41]. As we all know, β-tubulin is very stable in most cases, therefore, it is often used as reference gene. However in this study, we found the expression of β-tubulin obviously decreased after LPS stimulation according to the analysis of proteome. Chakravortty et al. reported that LPS can cause the disorganization of actin, tubulin and vimentin in bovine aortic endothelial cells [42], which is in concordance with our result. It is well-known that the intracellular target of cAMP is the cAMP-dependent protein kinase (PKA). The inactive PKA holoenzyme is a tetramer consisting of two regulatory (R) and two catalytic (C) subunits; and PRKAR2A is cAMP-dependent protein kinase type II-alpha regulatory subunit [43]. Our results of proteome analysis and verified by western blot revealed that the protein expression of PRKAR2A was extremely decreased after LPS stimulation.

WAT, as an important endocrine organ, plays a vital role on the regulation of inflammation by secreting various adipokines. However, our results of proteome analysis did not indicate that adipokines were regulated. Thus, we selected three of the most studied adipokines, namely leptin, adiponectin and ZAG, to investigate the contribution of WAT to systemic inflammation. The primary role of leptin is control of appetite while it also has an effect on regulating immunity [44, 45]. It can protect T lymphocytes from apoptosis, regulates T-cell proliferation and influences cytokines production from T lymphocytes [46]. Gualillo et al. found that inflammation can induce elevation of plasma leptin concentration in rats [47]. Accordingly, Sarraf et al. demonstrated that after administration of Escherichia coli LPS, leptin gene expression and leptin levels were increased [48]. However, our results revealed no difference in leptin expression after LPS stimulation. The reason may be the time of sampling and the dose of LPS stimulation. Adiponectin is best known for its role in the regulation of insulin sensitivity [49]. Recently, scientists demonstrated that adiponectin has the function of anti-inflammation as it can reduce the production and activity of TNF-α and IL-6 [50]. Besides, it can also induce the anti-inflammatory cytokines IL-10 and IL-1 receptor antagonist [51–53]. This may contribute to overcome inflammation in an infected organism. In our study, the expression of adiponectin increased under the condition of inflammation. Iwasa et al. found that serum adiponectin levels significantly increased at 6 h or 24 h after the injection of LPS [54], which is consistent with our results. ZAG is another adipokine and its overexpression occurs in all kinds of malignant tumors [55], thus, it is now recognized as a cancer marker. However, the biological functions of ZAG are largely unknown. According to the recent researches, ZAG can induce lipolysis by activating hormone-sensitive lipase and its activity is mainly mediated via the β_3_ adrenoreceptor with up-regulation of the cAMP pathway [56]. Its effect on inflammation is still widely unclear. In 2005, Boat et al. found that LPS can significantly increase the gene expression of ZAG in human adipocytes at the doses of 10 ng/mL and 100 ng/mL [57]. In the present study we found ZAG protein expression increased after LPS stimulation suggesting an association with inflammation of adipose tissue.

## Conclusion

LPS injection caused acute inflammation response in white adipose tissue. Most of lipid metabolism related genes were significantly influenced and 47 proteins demonstrated 2-fold changes in white adipose tissue in the condition of acute inflammation. Simultaneously, two adipokines, adiponectin and ZAG protein content were significantly increased. The results provide new clues to understand the adipose dysfunction during inflammation.

## Materials and methods

### Ethics statement

The experiment was conducted following the guidelines of Animal Ethics Committee at Nanjing Agricultural University, China. The euthanasia and sampling procedures complied with the “Guidelines on Ethical Treatment of Experimental Animals” (2006) No. 398 set by the Ministry of Science and Technology, China and “the Regulation regarding the Management and Treatment of Experimental Animals” (2008) No. 45 set by the Jiangsu Provincial People’s Government.

### Animals and experimental design

Twelve Duroc × (Landrace × Large White) male pigs from Huaian commercial farm of similar age and weight (12 ± 0.5 kg) were used in this experiment. The pigs were randomly divided into a control group and a lipopolysaccharide (LPS) injected group. They were fed thrice a day with a commercial pig diet for growing pigs. Water was available ad libitum. This study was conducted at the Jiangsu Academy of Agricultural Sciences.

Before starting the experiment, all the pigs were allowed to adapt to the environment for one week. After adaptation, the pigs of the LPS group (*n* = 6) were injected intramuscularly with LPS (E. coli serotype, Sigma Aldrich Ireland, Ltd) at a dose of 15 μg/kg body weight. The remaining pigs (*n* = 6) received an equivalent volume of physiological saline at the same time. Six hours after LPS injection, the pigs were sacrificed by exsanguination. Back fat samples were collected and frozen in liquid nitrogen, finally stored at -70 °C.

### RNA isolation, cDNA synthesis and real-time PCR

Total RNA was extracted from back fat samples with Total RNA Isolation Reagent (3101-100, Shanghai Pufei Biotech). Quantity of the RNA was measured by NanoDrop ND-1000 spectrophotometer (Thermo, USA). The ratios of absorption (260/280 nm) of all samples were between 1.8 and 2.0. Aliquots of RNA samples were subjected to electrophoresis using a 1.4 % agarose–formaldehyde gel to verify RNA integrity. Two micrograms of total RNA were treated with RNase-free DNase (M6101, Promega, USA) and reverse-transcribed according to the manufacturer’s instructions. Two microliters of diluted cDNA (1:20, vol/vol) were used for real-time PCR which was detected in Mx3000P (Stratagene, USA). All the primers were designed with the software “Primer Premier 6” and then blasted in NCBI. Peptidylprolyl isomerase A (PPIA), which is not affected by the experimental factors (LPS), was chosen as the reference gene. All the primers chosen to study the expression of genes related to immune response and lipid metabolism, as listed in Table 2, were synthesized by Generay (Shanghai, China). The method of 2^−ΔΔCt^ was used to analyze the real-time PCR results and gene mRNA levels were expressed as the fold change relative to the mean value of control group (Livak and Schmittgen, 2001).

**Table 2:** Primer sequences for real-time PCR amplification

Target genes	Reference/GenBank accession	Primer sequences
TLR2	NM_213761.1	F: GACACCGCCATCCTCATTCT R: CTTCCCGCTGCGTCTCAT
TLR4	AB188301.2	F: TCTACATCAAGTGCCCCTAC R: TAAATTCTCCCAAAACCAAC
NF-κB p65	KC316023.1	F: GGGGACTACGACCTGAATGC R: CACGGTTGTCAAAGATGGG
TNF-α	X57321.1	F: CCACGCTCTTCTGCCTACTGC R: TCGGCTTTGACATTGGCTACAA
IL-1α	NM_214029.1	F: TACTGACTATGGCTACCAA R: ATTCCAGCTGCTATTGTG
IL-1β	AY291592.1	F: CCGCCAAGATATAACTGAC R: GCAGCAACCATGTACCAA
IL-6	AF518322.1	F: AATGCTCTTCACCTCTCC R: CACACTTCTCATACTTCTCAC
ACACA	NM_001114269.1	F: GGCCATCAAGGACTTCAACC R: ACGATGTAAGCGCCGAACTT
FASN	NM_001099930.1	F: GTCCTGCTGAAGCCTAACTC R: TCCTTGGAACCGTCTGTG
SCD	NM_213781.1	F: CTACACAACCACCACTACCATCAC R: GCAAACGCCCAGAGCAAGG
HSL	HM591297.1	F: ACCCTCGGCTGTCAACTTCTT R: TCCTCCTTGGTGCTAATCTCGT
ATGL	EU373817.1	F: ACCTGTCCAACCTGCTGC R: GCCTGTCTGCTCCTTTATCCA
CPT-1A	NM_001129805.1	F: ACAACGAGGTCTTCCGAT R: AACGCAAAACCACCAAACCC
UCP2	NM_214289.1	F: GACGCCTACAAGACCATC R: CTCAGCACAGTTGACAATG
UCP3	NM_214049.1	F: GACGATGGATGCCTACAG R: CACCTTCTCCTTGATGACA
11β-HSD1	NM_214248.1	F: CCATGCTGAAGCAGAGCAAC R: AAGAACCCGTCCAGAGCAAA
ZAG	XM_003124307.2	F: GCAGCCGTGAACACCAAGC R: GGTACCTCCGCAGCATCCC
adiponectin	EF601160.1	F: CCGTTCAGCATTCAGTGT R: CAGCCTTGTCCTTCTTGTA
leptin	NM_213840.1	F: CATCCATTGTTCGCTGTG R: CTGTCCTCTCCATTAGTCTC
PPIA	JX523418.1	F:GACTGAGTGGTTGGATGG R:TGATCTTCTTGCTGGTCTT

*TLR2* toll-like receptor 2, *NF-κB p65* nuclear factor-kappa B p65, *TNF-α* tumor necrosis factor-α, *IL-1α* interleukin-1α, *ACACA* acetyl-CoA carboxylase alpha, *FASN* fatty acid synthase, *SCD* stearoyl-CoA desaturase, *HSL* hormone-sensitive lipase, *ATGL* adipose triglyceride lipase, *CPT-1A* carnitine palmitoyltransferase 1A, *UCP2* uncoupling protein 2, *11β-HSD1* hydroxysteroid (11-beta) dehydrogenase 1, *ZAG* zinc-α2-glycoprotein, *PPIA* Peptidylprolyl isomerase A

### Adipose tissue preparation, total protein extraction and western blot

Frozen back fat samples (400 mg each) were minced and homogenized in 2 mL each of ice-cold homogenization buffer RIPA containing the protease inhibitor cocktail (Complete EDTA-free, Roche, Penzberg, Germany). Protein concentration was determined using a BCA Protein Assay kit (Pierce, Rockford, IL, USA). Forty micrograms of protein extract from each sample were then loaded onto 7.5 % SDS-PAGE gels and the separated proteins were transferred onto nitrocellulose membranes (Bio Trace, Pall Co, USA). After transfer, membranes were blocked for 2 h at room temperature in blocking buffer and then membranes were incubated with the primary antibodies, in dilution buffer over night at 4 °C. After several washes in tris-buffered-saline with Tween (TBST), membranes were incubated with the secondary antibodies in dilution buffer for 2 h at room temperature. After several washes, bands were visualized by enhanced chemiluminescence substrate (Super Signal West Pico, Pierce, USA), and the signals were recorded by an imaging system (Bio-Rad, USA), and analyzed with Quantity One software (Bio-Rad, USA).

The primary antibodies were against adiponectin (Bioworld, BS2833, 1:1000), zinc-α2-glycoprotein (Santa Cruz, sc-21721, 1:500), TUBB (Bioworld, BS1482MH, 1:10000), and PRKAR2A (Bioworld, BS1929, 1:1000). The secondary antibodies were goat anti-rabbit horseradish peroxidase (HRP) antibody (Bioworld, BS13278, 1:10000) and goat anti-mouse HRP antibody (Bioworld, BS124789, 1:10000).

### Lipolytic enzymes activity assay

A modification of the procedure of Zhang et al. was used to extract and assay the lipolytic enzymes [58]. Briefly, 500 mg of frozen adipose tissue in 1 ml of homogenization buffer (0.1 M K ^+^-PBS containing 1 mM MgCl_2_, 1 mM DTT and 1 mM EDTA) was homogenized on ice for 30 min, and then centrifuged at 12,000 × g at 4 °C. The protein content of supernatants was determined using a BCA Protein Assay kit (Pierce, Rockford, IL, USA). Triolein without glycerin was used as substrate which can be hydrolyzed to glycerol by lipolytic enzymes. The supernatant together with prepared triolein were incubated for 1 h at 37 °C. The lipolytic enzymes in supernatant activate the lypolytic degradation of the triolein emulsion. The released glycerol was determined using a commercial kit (Applygen, China). Standard curves were constructed with pure enzymes to calculate the activities of the enzymes. All samples were measured in duplicate at appropriate dilutions.

### Radioimmunoassay

The content of leptin in back fat was measured using a commercial multispecies radioimmunoassay (RIA) kit (Beijing North Institute of Biotechnology, Beijing, China), according to the manufacturers’ instructions. A 200 mg frozen back fat sample was minced and homogenized in 1 mL phosphate buffered saline (PBS). Then, the homogenate was freeze-dried and redissolved in 200 μL PBS. Finally, leptin content was measured in this 200 μL solution.

### Label-free quantitative proteomics analysis

Another 200 mg back fat sample was weighed and the total protein was extracted using 1 mL ice-cold homogenization buffer RIPA containing the protease inhibitor cocktail (Complete EDTA-free, Roche, Penzberg, Germany). The same quality (250 μg) for each sample was used for the proteomics analysis. Next, the total protein was digested according to the method of filter-aided sample preparation (FASP) which combines the advantages of in-gel and in-solution digestion for mass spectrometry–based proteomics [59]. Then, LC-MS/MS method was used to quantify the peptide fragments [60]. This experiment was conducted in College of Life Science in Nanjing Agricultural University. Finally, the results were analyzed by employing the software MaxQuant [61]. The proteins which exhibited a change of at least 2-fold between groups were selected. The data analysis of the proteome was conducted by BioNovoGene. By using the software MaxQuant, the number of proteins identified for each animal in the control group was 817, 610, 616, 679, 575, and 829, respectively; and the number of proteins identified for each animal in the LPS group was 446, 712, 549, 756, 533, and 744, respectively. Then, according to the *P*-value < 0.05 and fold change > 2 after T test, we identified 47 regulated proteins.

### Statistical analysis

All data are presented as the means ± standard errors. The statistical analyses were performed using the Statistical Program for Social Sciences (SPSS) software, version 20.0, for Windows. The differences were tested via an analysis of variance (ANOVA), and a t-test was used for independent samples. Differences with P values < 0.05 were considered statistically significant.

### Consent

Written informed consent was obtained from the patient for the publication of this report and any accompanying images.

## Additional files

Additional file 1: Figure S1. The volcano plot of distinct proteins in label-free proteomics analysis. Additional file 2: Figure S2. The radar chart of distinct proteins in label-free proteomics analysis.

## Abbreviations

WAT: White adipose tissue
LPS: Lipopolysaccharide
TLR2: Toll-like receptor 2
NF-κB p65: Nuclear factor-kappa B p65
TNF-α: Tumor necrosis factor-α
IL-1α: Interleukin-1α
ACACA: Acetyl-CoA carboxylase alpha
FASN: Fatty acid synthase
SCD: Stearoyl-CoA desaturase
HSL: Hormone-sensitive lipase
ATGL: Adipose triglyceride lipase
CPT-1A: Carnitine palmitoyltransferase 1A
UCP2: Uncoupling protein 2
11β-HSD1: Hydroxysteroid (11-beta) dehydrogenase 1
ACSS2: Acyl-CoA synthetase short-chain family member 2
ACSBG2: Acyl-CoA synthetase bubblegum family member 2
ELOVL5: ELOVL fatty acid elongase 5
PRKAR2A: cAMP-dependent protein kinase type II-alpha regulatory subunit
TUBB: β-tubulin
ZAG: Zinc-α2-glycoprotein
PPIA: Peptidylprolyl isomerase A
