# Supplementary figures and images for: Lipopolysaccharide challenge significantly influences lipid metabolism and proteome of white adipose tissue in growing pigs
Source: Lipids Health Dis. 2015 Jul 8;14:68. doi: 10.1186/s12944-015-0067-5 (PMC4493945; doi:10.1186/s12944-015-0067-5)

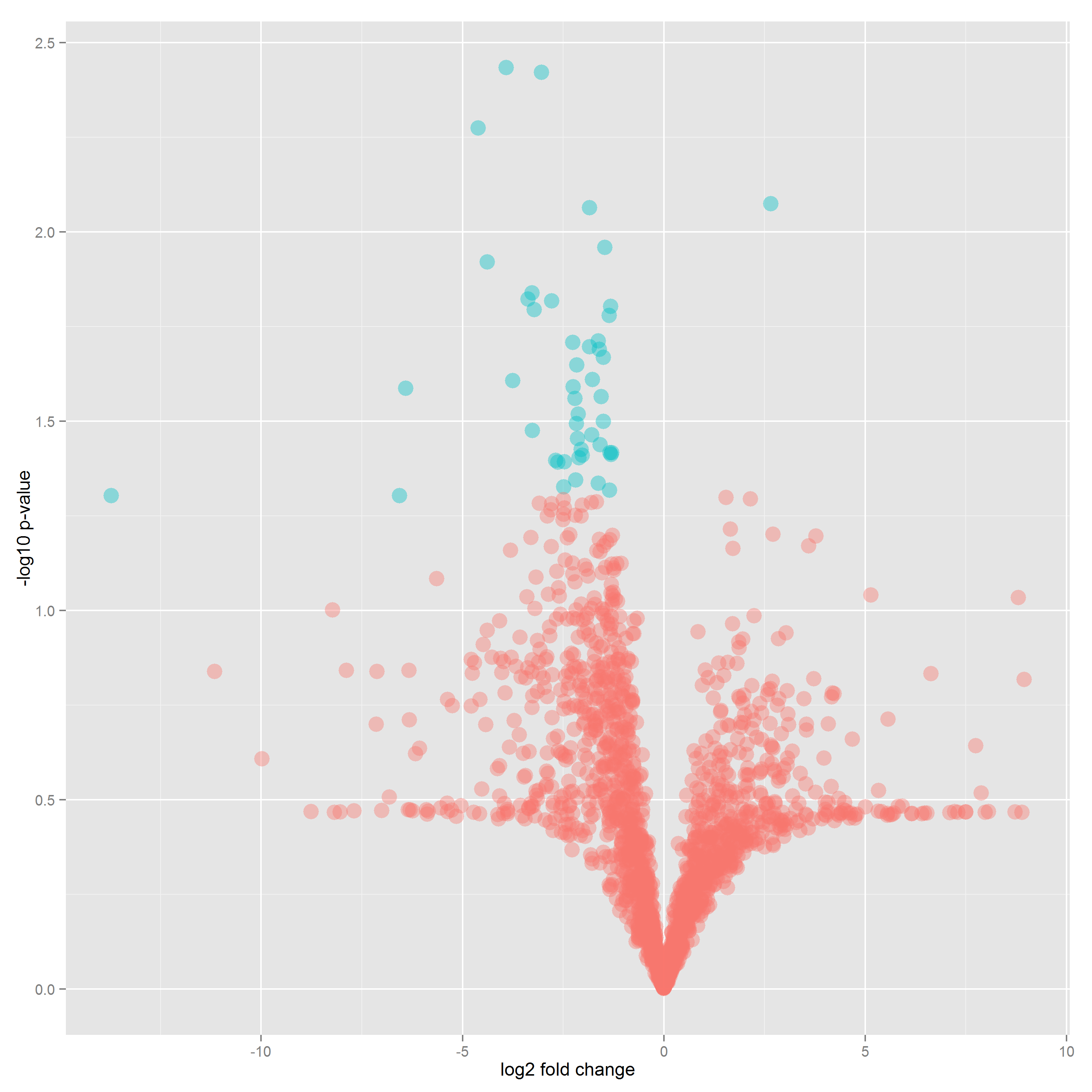

Supplement: Additional file 1: Figure S1. — The volcano plot of distinct proteins in label-free proteomics analysis. [file 12944_2015_67_MOESM1_ESM.png]

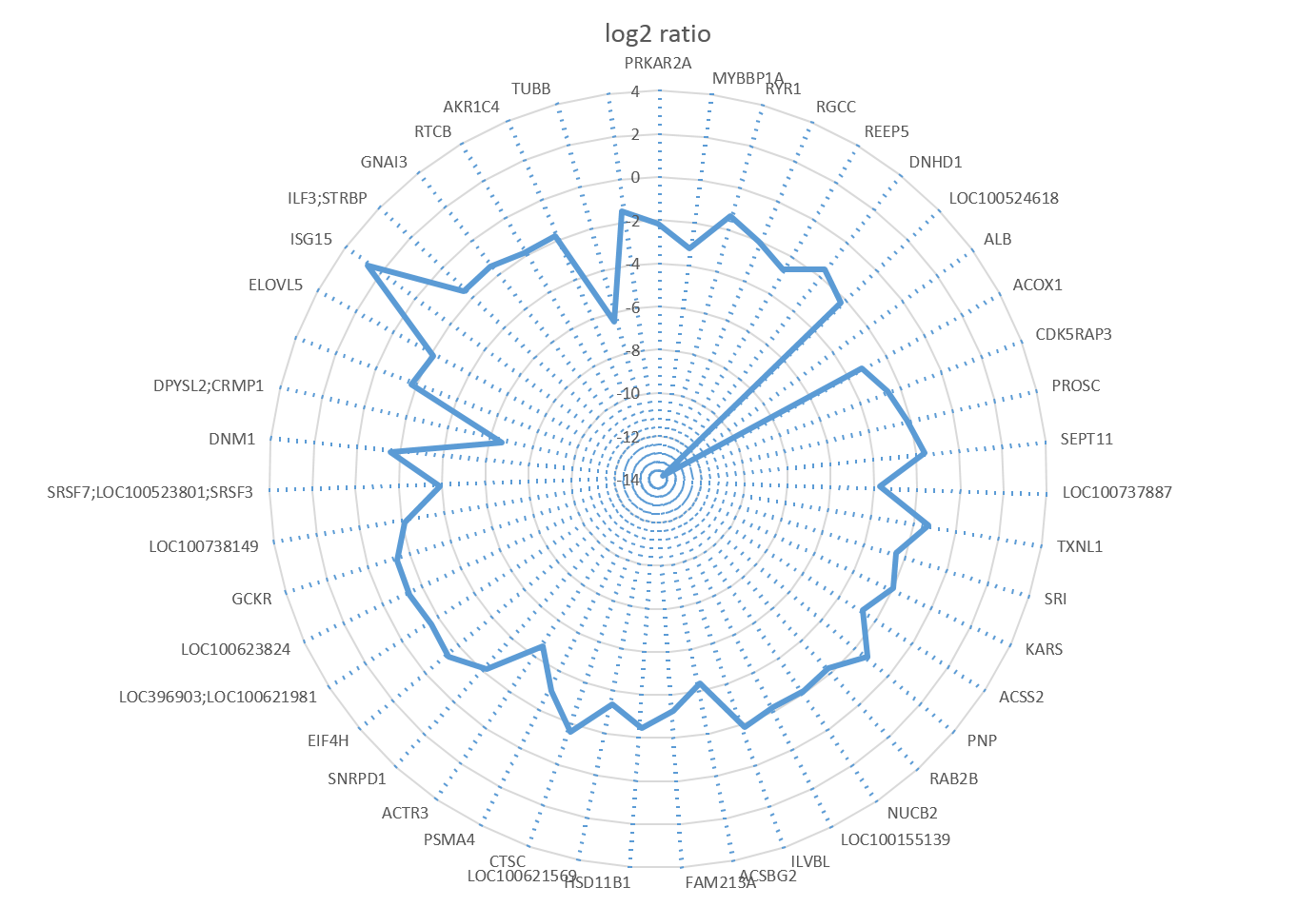

Supplement: Additional file 2: Figure S2. — The radar chart of distinct proteins in label-free proteomics analysis. [file 12944_2015_67_MOESM2_ESM.png]
